# Extending Research on Deception in Sport – Combining Perception and Kinematic Approaches

**DOI:** 10.3389/fpsyg.2019.02650

**Published:** 2019-11-28

**Authors:** Josefine Panten, Florian Loffing, Joseph Baker, Jörg Schorer

**Affiliations:** ^1^Institute of Sport Science, Carl von Ossietzky University Oldenburg, Oldenburg, Germany; ^2^School of Kinesiology and Health Science, York University, Toronto, ON, Canada

**Keywords:** deception, kinematics, expertise, dyad, interaction

## Abstract

The spatio-temporal demands of many high performance sport contexts require a strategic interplay between anticipation from early kinematic cues and the appropriate movement strategy. Despite the importance of the interaction between observer and deceiver in these contexts, this dyad is usually considered separately (i.e., from perceptual-cognitive or kinematic perspectives). The present approach proposes a consolidation of perceptual-cognitive and kinematic perspectives into a dyad of deception that focuses on the interplay between opposing actors within antagonistic contexts. A framework is proposed for analyzing movement deception within this dyad. Applying a functional approach, the deceptive act is positioned as a means of optimally solving an antagonistic performance task with high spatio-temporal demands. The framework involves three elements: first, the context of the movement deception is evaluated relative to the constraints imposed by the athlete, object, and deceptive content. Together, these constraints generate a range of potential kinematic options for movement deception. Second, movement deception is determined by the spatio-temporal constraints of the original context. More simply, misleading information is only useful if it mimics elements of the genuine movement. Third, the framework emphasizes targeting the spatio-temporal interplay as well as differentiating between active and co(ntra)-active movement deception. Our goal with this framework is to supplement movement deception research by providing a conceptional context that can be applied across sports.

## Introduction

The capacity to convey and infer intentions based on nonverbal information is a fundamental element of social interaction (Iacoboni et al., 2005). However, in some social situations, deceptive movements are used to mislead, by “intentionally causing another person to have or continue to have a false belief” (Mahon, 2007, p. 189). Over the past decade, researchers have emphasized that this strategy has particular significance for gaining an advantage in sports with high spatio-temporal constraints. Research highlights skilled athletes’ capacity to anticipate domain-specific action outcomes; they are both more accurate and more rapid than less skilled athletes in predicting movements from early kinematic cues.

However, despite this enhanced anticipation skill, they are susceptible to deception, perhaps more so than lesser skilled athletes (for reviews cf. Mann et al., 2007; Güldenpenning et al., 2017). In their analysis on deception detection, Jackson et al. (2006) noted two types of deceptive movement strategies: (1) actively providing misleading (deception) information and (2) withholding information that might provide critical cues for anticipation (disguise). This differentiation was grounded in a perceptual-cognitive perspective of expert performance. In this paper, we attempt to extend this discussion by integrating a kinematic perspective to reflect the complexity of the observer-deceiver dyad.

## Perspectives on Movement Deception

### The Dyad of Deception

**Figure 1:**
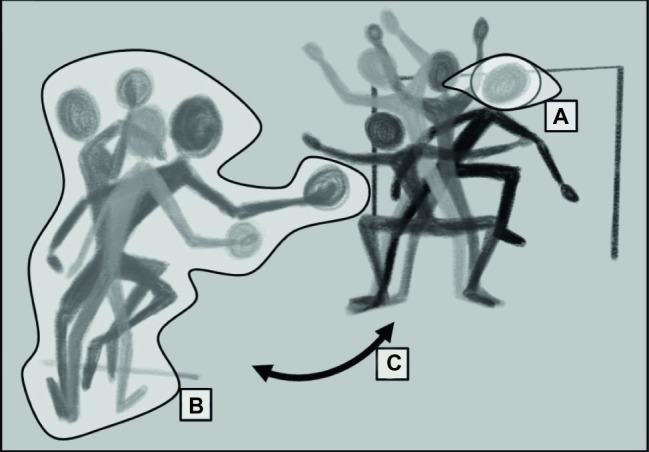
Dyad of deception with perceptual-cognitive **(A)**, kinematic **(B)**, and kinematic system interaction **(C)** components.

In essence, movement *deception*, defined as actively providing information that “misleads or ‘fools’ an observer into making an incorrect judgment” (Jackson et al., 2006, pp. 356–357), describes a complex and subtle communication between two actors. In this “dyad of deception” (Figure 1), both actors continually share information *via* the integrated processes of perception and movement. The deceptive act emerges out of the informational exchange between an actor intending to deceive (i.e., the deceiver) and a respective addressee (i.e., the observer). Inclusion of both elements of this dyad is important. For instance, while a range of methodological approaches have been advocated to improve ecological validity, such as *in situ* designs (Alaboud et al., 2016; Güldenpenning et al., 2018), virtual reality or animated research (Brault et al., 2012; Helm, 2016) or instructed interactions (Brault et al., 2010; Lopes et al., 2014; Helm et al., 2017), these designs end up decoupling movement (stimuli) from perception (task). Because of the critical links between perception and action (Gibson, 1979), concurrent examinations of deceiver and observer are needed in order to model the interplay of temporal and spatial factors between the performers. Nonetheless, research to date has contributed valuable insights into our understanding of movement deception, although this has been done by focusing on the perceptual-cognitive components (Figure 1A) or movement kinematics (Figure 1B) separately.

### Perceptual-Cognitive Component

Previous perceptual-cognitive research studies have provided evidence on the unilateral effects of movement deception in the observer (Figure 1A). In these studies, deception is measured by prediction accuracy and response time in observers judging binary coded outcomes or intention of movement deception. Results indicate that while performance is still superior to novice observers, experts are susceptible to deception, as highlighted in studies of soccer (Smeeton and Williams, 2012; Wright et al., 2013), tennis (Rowe et al., 2009), rugby (Mori and Shimada, 2013), and basketball (Sebanz and Shiffrar, 2009; Kunde et al., 2011). Importantly, these studies are framed using cognitive and perceptual theories (cf. Prinz, 1990; Hommel et al., 2001; Rizzolatti, 2005; Mann et al., 2007; Schütz-Bosbach and Prinz, 2007; Grafton, 2009; Huys et al., 2009), which position perceptual and motor expertise in terms of the expert’s response to respective stimuli in the performance environment (Cañal-Bruland et al., 2010; Güldenpenning et al., 2015). In this view, contextual factors such as the frequency and sequence of the deceptive stimuli affect susceptibility due to a learning bias of repeatedly used misinformation (Alaboud et al., 2012; Güldenpenning et al., 2018). Deciphering deceptive and non-deceptive movements is suggested to be cue-based (Jackson et al., 2006; Smeeton and Williams, 2012) and triggered through action observation and social networks (Bishop et al., 2013; Wright et al., 2013; Wright and Jackson, 2014). For a review on perceptual-cognitive effects of deception, see Güldenpenning et al. (2017).

### The Movement Kinematics Component

In addition to the perceptual-cognitive studies noted above, a small number of studies have examined the kinematics of movement deception (Figure 1B; Brault et al., 2010; Lopes et al., 2014; Helm et al., 2017). Results indicate that although the movements are highly complex, the deceptive act follows a generalizable structure, reflecting a balance between deceptive and genuine signals (Brault et al., 2010). More specifically, these studies suggest a range of functionality to which the movement deception has to correspond, and predefined deceptive stimuli (e.g., by explicit criteria) or binary categories (i.e., deceptive or non-deceptive movement) may oversimplify the matter. Relying on stimuli that lack related kinematic information (e.g., Rowe et al., 2009; Cañal-Bruland et al., 2010; Smeeton and Williams, 2012; Mori and Shimada, 2013) or are artificially generated from non-deceptive movements (e.g., Güldenpenning et al., 2013; Tomeo et al., 2013) may only provide part of the picture.

### Kinematic System Interaction

Considering the influence of kinematic information in movement deception may provide important information regarding the interaction between deceiver and observer (Figure 1C). Importantly, this interplay does not correspond to the unilateral differentiation suggested by the terminology; both actors can be *observer* and intentional *deceiver* at the same time. Thus, for understanding movement deception, the kinematics of the deceiver and the observer have to be set in spatio-temporal relation to each other. As a result, the functionality of a deceptive movement is dependent on its temporal and spatial execution in the context of each actor’s movements. This bilateral simultaneous exchange requires broadening our perspective of movement deception in a 1:1 situation: While operating within the rules for attacking or defending, both actors may deceive, yet the degree of influence over the situation differs. The deceiver may be active in the sense of an attacking player directly influencing the situation, or co(ntra)-active in the sense of a defending player indirectly influencing the situational outcome. For instance, in a soccer penalty situation, the penalty taker has direct influence on the situation by having to kick the ball, yet the goalkeeper may induce a directional bias by shifting their position between the goal posts.

Acknowledging the key roles of both perception and action, the section below explores a framework for movement deception from both perspectives. By addressing (1) situational constraints originating from the respective movement task, (2) kinematic requirements, and (3) the interactive deceptive dyad, the approach helps to characterize movement deception using a general approach that can be applied across sporting contexts.

## A Framework of Movement Deception From a Kinematic Perspective

Detached of intention, movement deception represents functional motor coordination in a given sporting context (Torres, 2000; Schorer et al., 2007). In its simplest form, motor execution reflects the means to solve a given task where movement is required (Göhner, 1979). Variation in the movement demands needed to “solve the task” can generally be explained *via* a range of diverse internal and external influencers (e.g., Göhner, 1979; Newell, 1986). For example, task goals, environmental affordances (e.g., weather), policies related to acceptable behavior (e.g., player conduct in team sports), and available equipment characterize the “rules” to which a functional movement has to adhere. Accordingly, for a kinematic movement analysis, the kinematics as well as the respective constraints are considered (for translation, see cf. Hossner et al., 2015).

In the present approach, the movement is both a means to solve a given task like scoring a goal against a goalkeeper and to intentionally mislead another person (i.e., the goalkeeper). The following framework evaluates both the *constraints of the task* (section “Constraints in Movement Deception”) and the *kinematic components* (section “Kinematics of Movement Deception”) to determine how they inform and limit movement deception. Critically, this perspective is integrated in a *dyad* (section “Dyadic Active and Co(ntra) – Active Movement Deception”) led by the assumption that deception evolves out of the informational exchange between deceiver and observer.

### Constraints in Movement Deception

**Figure 2:**
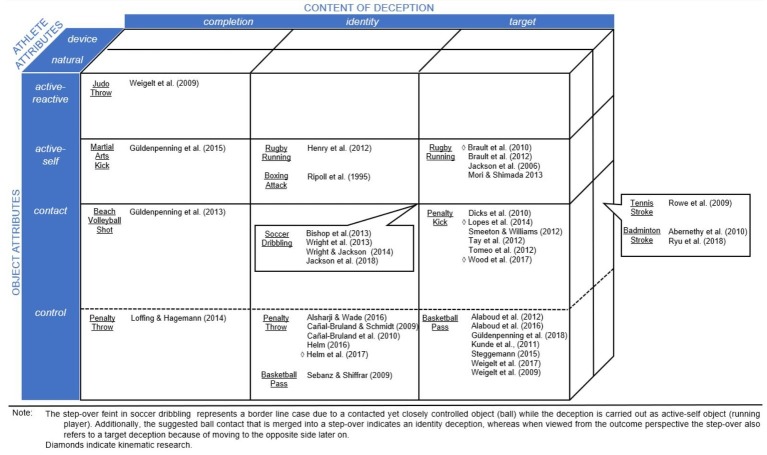
Categorization of researched movement deception.

To date, the situations covered by sport-related deception research include penalty situations (Smeeton and Williams, 2012; Loffing and Hagemann, 2014), on field duels (Mori and Shimada, 2013), martial art settings (Ripoll et al., 1995; Güldenpenning et al., 2018), and racquet sports (Jackson et al., 2018; Ryu et al., 2018) as summarized in Figure 2. At the most basic level, the functionality of deception is dependent on constraints that characterize the respective task: *goal*, *environment*, *rule*, *object*, *athlete*, and *device* (Göhner, 1979).

For movement deception, the movement goal has to meet two objectives in order to be functional: to execute a movement that is capable of achieving the intended movement goal and to feign a different outcome in order to mislead the opponent. In terms of the misleading goal, the content may differ, for example, deceiving about the target, using ambiguous movements or feigning the execution. In addition, other factors can impose constraints on the movement deception kinematics and help to differentiate actions across sports, such as the characteristics of the athlete, the device used (e.g., racquet) and the object that is being manipulated (e.g., ball, puck, etc.). In the following section, the movement deceptions examined in previous research are categorized by constraints imposed by the *athlete* (section “Athlete Attributes: Natural and Device Supported”), the *object* (section “Object Attributes and Respective Relations”) and the *content* (section “Content of Deception”) (see Figure 2). Note, environment (e.g., field and pitch) and game rules are specific to each sport and, thus, are excluded in the interest of a more general framework on movement deception.

#### Athlete Attributes: Natural and Device Supported

Athlete characteristics, along with any equipment used, constrain the range of available sport movements (Göhner, 1979) by defining the level of control within the movement system.

The athlete can be classified as *natural*, exploiting the body alone, or *supported by a device*. Although skilled athletes essentially integrate sport devices (e.g., racquet and glove) as part of their body, the device remains an external tool potentially reducing control and feedback when compared to a natural athlete. For movement deception intended to mislead the observer *via* kinematic information, such reduction of control would be crucial, considering that the device normally constitutes the link between athlete and object (e.g., ball) in achieving the movement task.

The majority of research has focused on natural athlete’s deception, such as in studies of rugby (Brault et al., 2012; Mori and Shimada, 2013), penalties in soccer and handball (Cañal-Bruland et al., 2010; Smeeton and Williams, 2012; Loffing and Hagemann, 2014), volleyball (Güldenpenning et al., 2013), martial arts (Ripoll et al., 1995; Weigelt et al., 2009), and passing in basketball (Steggemann, 2015; Alaboud et al., 2016; Weigelt et al., 2017). Device supported examples of movement deception research, however, are scarce and restricted to badminton (Abernethy et al., 2010; Ryu et al., 2018) and tennis (Rowe et al., 2009) (see Figure 2).

#### Object Attributes and Respective Relations

Object attributes also affect the level of control and range of functionality when inducing misleading information. In this framework, the object refers to the central implement that has to be moved purposefully for achieving the sport’s aim. Its characteristics influence the movement and the relation between object and athlete (e.g., hand size in relation to ball size influences the level of control), while additionally refining the movement constraints (Göhner, 1979). Functional movement deception takes place within the range defined by object attributes. More specifically:

1. In *active-self*, athletes serve as objects resulting in direct control during movement. For movement deception, the time between deceptive and genuine actions is variable in temporal sequencing. However, often these actions are restricted by spatio-temporal constraints related to global stability (e.g., in rugby side step feints; Jackson et al., 2006; Brault et al., 2010; Henry et al., 2012; Mori and Shimada, 2013) and involve comparatively salient, but balanced cues *via* whole-body deception (Sebanz and Shiffrar, 2009; Brault et al., 2010).
2. *Active-reactive* is similar to the category above but in antagonistic dyads, where objects move in a dynamic interaction with another object/performer such as in most combat sports. For a movement deception to be functional, it requires handling the opponent in a way that he or she is deceived about their own movement trajectory (e.g., judo; Weigelt et al., 2009). Consequentially, it entails severe constraints imposed by the availability of direct and multi-level information (e.g., sight, haptics, balance, acceleration, or pressure). Nonetheless, while the opponent uses multiple types of sensory information to aid more accurate judgments compared to relying on a single sensory system (e.g., vision), the situation allows deception through a range of spatio-temporal, haptic, and/or pressure related cues. Note, here the allocation of the attributes of active-self or active-reactive depends on the respective movement’s reference frame. For instance, in martial arts, the object is generally characterized as active-reactive aiming to achieve a score such as through execution of a successful technique. On the other hand, specific movements are considered comprising active-self characteristic, for example, the punch, kick, etc. (Ripoll et al., 1995; Hussein, 2004; Güldenpenning et al., 2015).
3. *Passive-reactive* objects are dependent upon the athlete to move. Evaluating the constraints imposed on the object during movement deception, a distinction is proposed between objects (a) controlled throughout or (b) contacted at a particular point in time within the movement.
  - *Controlled passive-reactive* objects, though indirectly controlled, share the characteristics of direct control of the active-self *via* the effectors. Changes in the outcome can be made for an extended time until release of the object, such as in handball penalty throws (Cañal-Bruland and Schmidt, 2009; Cañal-Bruland et al., 2010; Loffing and Hagemann, 2014; Helm, 2016) or basketball passing (Sebanz and Shiffrar, 2009). As effector specific movement outcomes, the constraints indicate less salient kinematic cueing (Sebanz and Shiffrar, 2009) when compared to whole body movement of active-self objects. Equally, effector-dependency allows for simultaneous inclusion of misleading social cues detached from goal-orientated movements such as gaze or head fakes (e.g., Kunde et al., 2011; Steggemann, 2015; Alaboud et al., 2016; Weigelt et al., 2017; Güldenpenning et al., 2018).
  - *Contacted passive-reactive* characterizes short object-athlete contact during which the effector transfers the force for the subsequent movement outcome. For deceptive movements, it entails the spatio-temporal limitation of constraining the resolution toward the genuine movement until the point of contact: independent of any deception before, at the point of contact the genuine intention has to be carried out. This is the case in badminton (Abernethy et al., 2010; Ryu et al., 2018) or tennis strokes (Rowe et al., 2009), soccer penalty kick (Dicks et al., 2010; Smeeton and Williams, 2012; Tay et al., 2012), or volleyball shot (Güldenpenning et al., 2013).

In sum, the range of deceptive movements resulting from object manipulation differs depending on the object and its respective relation to the athlete.

#### Content of Deception

In addition to the genuine movement goal (Göhner, 1979), a movement with deceptive intent imposes constraints that are supplementary to the motor demands of the movement. It requires suggesting an outcome different from the genuine one by incorporating both sufficiently within a functional movement trajectory (Runeson and Frykholm, 1983; Schnabel, 2007). The “suggestion” required for the deception can differ in terms of its content. In an initial discourse on strategies to impede anticipation, Schnabel (2007) refers to at least two possible solutions regarding what a deceptive movement could entail. First, the movement may be aborted all together, followed by a restart of a different movement. Second, one movement might be merged into a different one leading to an altered outcome.

This structural approach can be re-integrated into a content-related differentiation comprising constraints for movement deception kinematics. For instance, *completion deception* implies a complete termination of the movement designed to mislead the opponent before executing the actual intended movement. Examples are controlled passive-reactive basketball passes (Sebanz and Shiffrar, 2009) when a throw is initiated but stopped prior to ball release. Other examples are active-self rugby side step (Henry et al., 2012) or punch feints in boxing (Ripoll et al., 1995). Note, comparing Cañal-Bruland and respective colleagues (Cañal-Bruland and Schmidt, 2009; Cañal-Bruland et al., 2010) and Helm et al. (2017), who both investigated deceptions on handball penalty throw completion, nuances of completion become apparent. These range from completely aborting all induced forces of the movement to a mere abort of throw yet continued circular movement with a redirection of induced force.

Additionally, when athletes merge or evolve their movements in order to deceive, this is done in the content of target and identity elements. Deceptive movements often involve a conflict between competing action outcomes. Accordingly, the deceptive and genuine elements have to be convincingly combined rather than simply included as separate movement elements (i.e., as in completion deception). The content of the deceptive movement also differs in terms of usage of knowledge within the dyad.

In *identity* deception, sport-specific techniques are assimilated to create ambiguity. For instance, early kinematic cues suggest an action that could result in either a spike or a poke shot in a volleyball attack (Güldenpenning et al., 2013). The deceptive content is generated based on an observer’s experience having encountered both techniques as well as the techniques’ degree of similarity and probability (e.g., straight vs. roundhouse kick; Güldenpenning et al., 2015; direct vs. lobbed shot; Loffing and Hagemann, 2014).

*Target* deception, in contrast, may work with less sport-specific expertise, relying on directional judgment from kinematics. Often, opposite targets for active-self or passive-reactive objects are indicated in the course of a movement, for example, during rugby in running feints (Jackson et al., 2006; Brault et al., 2012; Mori and Shimada, 2013) and in soccer penalty kicks (Smeeton and Williams, 2012; Lopes et al., 2014).

As a constraint, the movement content of the deceptive action can differ relative to structural (separated or merged deceptive and genuine entities) and aspect-related (execution, technique, and direction) characteristics. The attributes of completion, identity, and target define a context out of which the functionality of the movement deception emerges (i.e., is the movement actually deceptive).

Collectively, the attributes of the athlete, object, and deceptive content define the range to which a functional deceptive movement has to correspond. Accordingly, the analysis of movement kinematics has to be interpreted with reference to relevant constraints.

### Kinematics of Movement Deception

Functional movement deception involves the negotiation of kinematic cues suggesting both deceptive and genuine intent while adhering to the constraints discussed above. Schorer et al. (2007) suggested deceptive movement expertise depends on a high degree of *functional movement variability* – meaning intentionally created variability – while ensuring a consistent outcome. For instance, their movement trajectory analysis revealed multiple clusters of skilled players’ penalty throw trajectories that were linked to certain targets. Specifically, skilled players performed multiple patterns for one target as well as one similar pattern targeting diverse areas in the goal. Given the instruction to throw imagining a goalkeeper, it was argued that athletes use variability to negotiate external perturbations (Müller and Loosch, 1999; Wilson et al., 2008) as well as an intentional functional tool to induce deceptive cues (Schorer et al., 2007).

For the kinematic structure of movement deception, the athlete’s ability of using functional movement variability suggests a near infinite number of kinematic movement patterns for movement deception. Yet, the resolution of the deception toward the intended outcome requires understanding the spatio-temporal constraints that shape the structure of a deceptive movement. These spatio-temporal constraints reflect a structure ranging from sequential to simultaneous order (Table 1).

**Table 1:** Categorization of structural characteristics of kinematic research.

Sequential	Simultaneous
Lopes et al. (2014) Helm et al. (2017) Brault et al. (2010)	Wood et al. (2017)

In a *sequential* order, deceptive and genuine elements precede each other before being resolved in the form of the intended outcome, such as in rugby running feints (Brault et al., 2012), lobbed handball throws (Loffing and Hagemann, 2014), throw terminations (Sebanz and Shiffrar, 2009) or badminton shots (Ryu et al., 2018).

In research exploring deception using a passive-reactive object, Lopes et al. (2014) investigated the soccer penalty in terms of the directional information available in the kinematics. Participants were asked to simulate – without further specification as to how – a kick to one side while shooting to the opposite goal side. Target relevant indications in the kinematics centered on the lower body with spatial and temporal proximity to the contact point. Deceptive elements were found in distant areas to the ball at ball contact and were more pronounced temporally distant to contact time. The findings are in line with the requirements of genuine movement components at ball contact.

For controlled passive-reactive objects in handball penalties, Helm et al. (2017) compared the trajectories of selected body parts in deceptive and non-deceptive throws. While the temporal course did not differ significantly, spatial analysis revealed differences in distal body parts specifically in non-throwing and throwing arm when recapturing the induced energy.

Evidence on the active-self object (i.e., athlete) in a whole body target deception is found in rugby side step running feints, as examined by Brault et al. (2010). Here, the final stable movement change comprises the genuine outcome, and deceptive (i.e., exaggerated) kinematics were found within distal areas of the body while central elements (e.g., lower trunk and center of mass) adhered to the genuine outcome. The time line indicated an interplay between temporal and spatial components: for overall stability, displacement of the center of mass was delayed to counterbalance the deceptive displacements involved with a whole body directional change.

In sum, evidence indicates deceptive movement kinematics and genuine kinematics differ. Irrespective of object attributes (controlled and contacted passive reactive or active-self), differences in movement trajectories were found distal (i.e., further away) to the included object (for active-self the center of mass applies as the reference for object location). Depending on the respective target or completion deception, the difference can be interpreted as misleading information or giving away the deceptive intent.

On the other end of the continuum, a *simultaneous* order of deception refers to an informational conflict imposed at the same time within movement execution. For instance, in a basketball pass “head fake” (e.g., Kunde et al., 2011; Steggemann, 2015), contradictory directional information is presented by the passer turning her head to one direction while passing to the other (e.g., head turned right, pass to the left).

Evaluating the costs of a simultaneous deception on contacted passive-reactive objects, Wood et al. (2017) investigated gaze fakes in soccer penalty shots. They found fixations to the opposite target location decreased shooting accuracy resulting in more centralized shots. This effect could only be counteracted by processing additional information in the form of the goalkeeper’s location (a critical strategy as described below). Although there were no kinematic analyses of the movement itself, findings emphasize the costs even non-movement-related deception (i.e., *via* gaze misdirection) can have on motor performance.

While there are studies looking at the kinematics of the deception, current research is limited by omitting previously outlined kinematic movement interaction within the dyad (Figure 1). As characterized in the notion of functional movement variability addressed earlier (Schorer et al., 2007), deceptive movements originate from uncertainty in the outcome. Contradicting this notion, current research on movement deception kinematics intends to identify deceptive versus non-deceptive patterns (differences between genuine and deceptive movements; Brault et al., 2010; Helm et al., 2017; origin of deceptive cues; Lopes et al., 2014) where – ideally – reliable patterns should not be present. Though present kinematic research wisely avoids including prescriptive patterns and formulates rather vague instructions such as “shoot to green but simulate shooting to red” (Lopes et al., 2014, p. 204) or “mimic a genuine throw without final ball release” (Helm et al., 2017, p. 301), this vagueness also leads to mixed interpretations on what kind of movement is actually investigated.

Equally, the success of the movement deception depends on the observer’s susceptibility to the generated trajectory. Thus, rather than finding a movement pattern, kinematic movement deception research should include the observer and consider deception as a situational interaction of movements. For example, research on team interactions has outlined the individual-environment relationship – comprising player-opponent dyad, object and pitch as key constraints – as an important factor in shaping sport performance (McGarry et al., 2002; Bourbousson et al., 2010; Vilar et al., 2014). Characterized by non-verbal communication between two actors, such approaches move the focus from movement pattern alone to the movement pattern’s meaning within this interactive communication process.

Therefore, the kinematic perspective we suggest considers both deceiver and observer equally relevant for deception – and requires concurrent kinematic analysis of both actors. Schnabel (2007) argued the interval to reorganize movement was 60–100 ms. Therefore, when considering both actors’ movement timelines, the effect of a deceptive movement may be related to a certain point in time (sequential deception) or throughout the complete movement (simultaneous deception) leaving an observer little or no time to adjust to the genuine objective. Accordingly, a *deadline hypothesis* could be formulated relative to the threshold at which the performer does not have enough time to adjust to the genuine objective. Research of the kinematic interplay of both actors is needed to identify the factors related to the successful execution of deceptive movements within each context’s specific temporal and spatial constraints.

### Dyadic Active and Co(ntra) – Active Movement Deception

Sport situations are generally described using clearly defined rules governing the behavior of active and passive actors (e.g., penalty taker and goalkeeper), generally reflecting a unilateral influence on the situation. Considering a communicative dyad, however, implies a bilateral and concurrent interaction which supports the distinction between active and co(ntra)-active movement deception in a kinematic framework. For instance, *active movement deception* corresponds to the common rule related description of an active actor with direct influence on the situational outcome (e.g., trick throws, running feints, and head fakes). Active movement deception has been the focus in the differentiation discussed in Figure 2.

Conversely, *co(ntra)-active movement deception* is based on an indirect influence on respective situations. It comprises movements concurrent with the actions of an active opponent likewise with the intent to induce misleading information. Thus, rather than just reacting to movements, the co(ntra)-active strategy adjusts the odds of a likely outcome. Though not restricted to its use alone, they often draw from spatial social cues such as gestures or posture. For instance, behavioral research into goalkeeping (Lobinger et al., 2014; Furley et al., 2017) highlights goalkeepers’ influence on penalty shooting performance. Influential cues identified for the penalties include the goalkeeper’s horizontal and vertical position relative to the goal or opponent, their posture (e.g., arms raised and foot placement) as well as specific (e.g., pointing) or unspecific gestures (e.g., waving) as referenced in Table 2. It is argued that these actions bias spatial judgments and guide attentional focus (Van der Kamp and Masters, 2008; Wood and Wilson, 2010; Weigelt et al., 2012; Kurz et al., 2018). Yet, inconsistencies in the results of in field and lab studies, as well as the limited scope of situations explored, require additional research on the topic.

**Table 2:** Categorization of researched co(ntra)-active movement deception.

			Soccer goalkeeping	Handball goalkeeping
Spatial cues	Gesture	Specific	Weigelt et al. (2012)	
		Unspecific	Wood and Wilson (2010) Furley et al. (2017) Kurz et al. (2018)	Lobinger et al. (2014)
	Presence	Posture	Weigelt et al. (2012)	Van der Kamp and Masters (2008) Lobinger et al. (2014)
		Position	Masters et al. (2007) Weigelt et al. (2012)	Lobinger et al. (2014)

The dyadic aspect we note above, where both partners influence the success of the deceptive act, has been acknowledged as being confounded by issues such as the need to match the expertise level of both deceiver and observer (e.g., Helm, 2016) and consider individuality (e.g., Lopes et al., 2014) as an important factor related to a performer’s susceptibility to deception. Brault et al. (2010, 2012) supplied a preliminary approach concerning the movement system when they investigated the kinematics of side-step running feints in rugby 1:1 duels determining the success of a deception based on the kinematic reaction of a defender (Brault et al., 2010). In a second study (Brault et al., 2012), they applied the findings on kinematics to a virtual reality setting investigating the interaction based on the defender’s response kinematics. Investigating the intercepting movement allowed them to determine the degree to which performers were deceived and the relation between the deceptive displacement and actor’s distance in the form of early movement bias. Still, communication between actors was omitted because actions were considered separately.

Collectively, a methodological approach that applies concurrent kinematic analysis of the dyadic movement system is needed to capture the communicative process that results in an observer being deceived. Prior kinematic studies and perceptual cognitive research have focused on the temporal and spatial appearance of deceptive information within a movement, overlooking that this information is ultimately integrated and interpreted *via* a dyad. The deceiver-opponent interplay, however, first confers meaning to the movement deception in the context of the whole kinematic system interaction. A crucial point for deceptive information to be induced in the interaction (e.g., *dead line hypothesis*; *just-in-time hypothesis*; Schorer, 2006) may relate to how the deception is built up over the time course. Such knowledge may aid research into perceptual-cognitive processes; for example, temporal occlusion could be adjusted to meaningful components rather than standardized time frames (e.g., Mori and Shimada, 2013; Loffing and Hagemann, 2014). In terms of the spatial distribution of information, concurrent kinematic analysis may aid our understanding of the coupling of movements, such as providing information regarding how to act on the generated reaction (e.g., causing the goalkeeper to shift the weight from one foot to the other, where the ball was thrown past).

## Closing Remarks

Deceptive movements pose a unique situation for understanding the nuances of high perceptual-cognitive and motor skill. While the perceptual-cognitive approach provides advances on anticipation of deceptive intent, kinematic research on this subject is limited. The framework presented in this paper supplements advances to the research field, providing a combined perspective of perception and kinematics in the form of a dyadic movement system interaction. Furthermore, in this framework, the focus is redirected toward deception originating from non-verbal communication in respective contexts rather than representing a unilateral action.

## Author Contributions

All authors listed have made a substantial, direct and intellectual contribution to the work, and approved it for publication.

### Conflict of Interest

The authors declare that the research was conducted in the absence of any commercial or financial relationships that could be construed as a potential conflict of interest.
